# Novel Processing Technology of Dairy Products

**DOI:** 10.3390/foods10102407

**Published:** 2021-10-11

**Authors:** Ekaterini Moschopoulou

**Affiliations:** Laboratory of Dairy Research, Department of Food Science and Human Nutrition, Iera Odos 75, 11855 Athens, Greece; catmos@aua.gr; Tel.: +30-210-529-4680

Milk has been processed into dairy products using traditional methods for hundreds of years.

However, research on new technologies in order to develop new products or to improve those already existing is constantly conducted. More specifically, modern processing approaches are used in order to change the texture, to improve the organoleptic properties, to ensure the safety, to extend the shelf life, and finally, to increase the nutritional and health value of dairy products. Pulsed Electric Fields, Ultrasound, and High-Pressure (HP) Processing are some of the novel technologies that are considered as alternatives to heat treatment. Among these, HP is the most promising technology during which the product is treated mainly in the range of 100–600 MPa at ambient temperatures; as a result, several constituents and properties of the treated product are altered [1]. Moreover, membrane technology is widely used in the dairy industry for separation or fractionation purposes, depending on the membrane pore size and the applied pressure. Microfiltration (MF), which involves membranes with a pore size of 0.1–10 μm, can remove bacteria and spores from skim milk; hence, it is also called ‘cold pasteurization’ [2]. In addition, apart from the novel technologies, modern analytical methods have been developed for the better determination or evaluation of significant characteristics and processing steps in the production of the dairy products.

The present Special Issue includes five research papers and two review papers. Four papers study the application of HP on milk and yoghurt products [3,4,5,6], one paper deals with the MF of milk [7], another one reviews the production of specific dairy ingredients [8], and the last one examines the use of modern analytical methods in the characterization of the recrystallization process in ice cream [9].

Briefly, Diez-Sánchez et al. [3] applied HP to produce milkshakes containing chokeberry pomace with the use of different high-pressure parameters and ratios of chokeberry. The researchers, with the aid of response surface methodology, concluded that a novel, highly nutritional product with increased antioxidant capacity and total phenolic content could be produced from milk that contains 10% (*w*/*v*) chokeberry and at a high pressure of 500 MPa for 10 min. The effect of HP on two probiotic microorganisms used in yoghurt production was studied by Tsevdou et al. [4]. These authors investigated different parameters and reported that HP in the range of 200–300 MPa improved the rheological properties of the product, while the viability loss of 0.5–1.2 logCFU/g remained constant during refrigeration storage. In addition, the same product enriched with a sweet cherry flavor behaved in the same way when treated with HP under the same conditions. Sakkas et al. [5] used HP as an alternative method for heating ovine milk enriched with whey protein concentrate (WPC) or whey protein concentrate hydrolysates (WPCH) in yoghurt production. WPCH had been developed by the same researchers by hydrolyzing WPC from Feta cheese whey with trypsin or Protamex. It was found that the heating method, i.e., conventional or HP, affected the yoghurt gel properties more than the enrichment with WPC or WPCH. More specifically, HP at conditions similar to heating caused inferior gel properties no matter the type of enrichment, and the addition of WPCH to skim ovine milk at a ratio of >0.5% dramatically worsened the gel properties. Wazed and Farid [6] developed a novel hypoallergenic product for infant nutrition. They applied HP to a reconstituted infant milk formula enriched with α-Lactalbumin, using different conditions of pressure, temperature, and time, and comparing it to pasteurization at 72 °C. The authors concluded that the highest ratio of α-Lactalbumin to β-Lactoglobulin level, which assure low allergenicity, was achieved after HP at 600 MPa, 40.4 °C, for 5 min. 

Regarding membrane technology, Panopoulos et al. [7], who applied cross-flow MF with the use of ceramic membranes with a pore size of 1.4 μm and a transmembrane pressure of 1.5 bar at 50 °C, reported that microbial flora reduction was higher in skim ovine milk (0.4% fat) than in skim bovine milk (0.3% fat). Moreover, although ovine and bovine permeates showed lower protein content than the respective unprocessed milks, the cheesemaking properties of ovine milk were not significantly affected. The authors concluded that the application of MF on ovine milk under the studied conditions can be used to treat this milk prior to cheesemaking. Huang et al. [8], reviewing the methods for producing dairy ingredients enriched in milk phospholipids (MPLs), reported that the membrane separation process as compared to methods involving the use of organic solvents—i.e., supercritical fluid extraction (SFE) by CO_2_ and dimethylether (DME), or SFE and DME, or organic solvent extraction—is the most efficient way to concentrate MPLs. Moreover, in this particular case, the carbon footprint of membrane technology is the lowest. 

As far as analyses are concerned, Kamińska-Dwórznicka [9] reviewed and compared modern methods for testing and describing the recrystallization of ice crystals, a process detrimental to the quality of ice cream products. They refer to methods such as microscopy and image analysis, focused beam reflectance measurement, oscillation thermo-rheometry, nuclear magnetic resonance (NMR), splat cooling assay, and X-ray microtomography; they concluded that all presented methods are suitable for describing the recrystallization process. However, only microscopy and image analysis can show both the changes in size and shape of ice crystals as well as their location. 

Finally, the editor of this Special Issue would like to thank the authors who submitted their papers and provided the readers with new information about high-pressure processing, membrane processing, and modern analytical methods for the study of the dairy products.
